# Supplementary material for: Comparative multiomics analysis of cell physiological state after culture in a basket bioreactor
Source: Sci Rep. 2022 Nov 23;12:20161. doi: 10.1038/s41598-022-24687-4 (PMC9686226; doi:10.1038/s41598-022-24687-4)

W_023666040.1:2182828:2186492:-@NW_023666040.1:2182828:2186733:-@NW_023666040.1:2208682:2208783

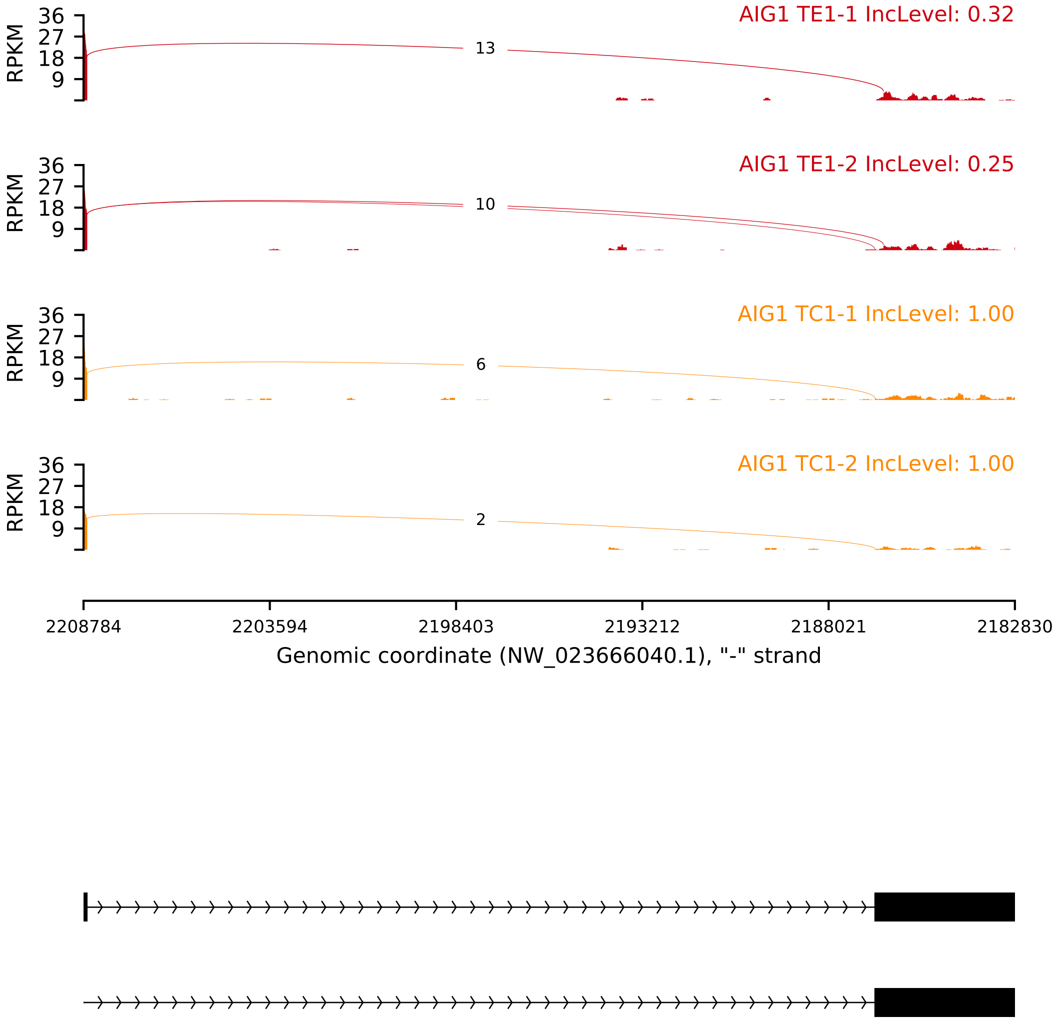

Supplement: Supplementary file 1 — Supplementary Information 1. [file 41598_2022_24687_MOESM1_ESM.zip › raw data/Transcriptomic raw data/7.AS/2.dasplot/TE1vsTC1/A3SS/3_AIG1_NW_023666040.1_2182828_2186492_-@NW_023666040.1_2182828_2186733_-@NW_023666040.1_2208682_2208783_-.pdf]
